# Rectal Swabs for Analysis of the Intestinal Microbiota

**DOI:** 10.1371/journal.pone.0101344

**Published:** 2014-07-14

**Authors:** Andries E. Budding, Matthijs E. Grasman, Anat Eck, Johannes A. Bogaards, Christina M. J. E. Vandenbroucke-Grauls, Adriaan A. van Bodegraven, Paul H. M. Savelkoul

**Affiliations:** 1 Department of Medical Microbiology and Infection control, VU University medical center, Amsterdam, the Netherlands; 2 Department of Gastroenterology and Hepatology, VU University medical center, Amsterdam, the Netherlands; 3 Department of Epidemiology and Biostatistics, VU University medical center, Amsterdam, the Netherlands; 4 Department of Internal Medicine, Gastroenterology and Geriatrics, ORBIS medical center, Sittard-Geleen, the Netherlands; 5 Department of medical microbiology, Maastricht University medical center, Maastricht, the Netherlands; New York State Dept. Health, United States of America

## Abstract

The composition of the gut microbiota is associated with various disease states, most notably inflammatory bowel disease, obesity and malnutrition. This underlines that analysis of intestinal microbiota is potentially an interesting target for clinical diagnostics. Currently, the most commonly used sample types are feces and mucosal biopsy specimens. Because sampling method, storage and processing of samples impact microbiota analysis, each sample type has its own limitations. An ideal sample type for use in routine diagnostics should be easy to obtain in a standardized fashion without perturbation of the microbiota. Rectal swabs may satisfy these criteria, but little is known about microbiota analysis on these sample types. In this study we investigated the characteristics and applicability of rectal swabs for gut microbiota profiling in a clinical routine setting in patients presenting with various gastro-intestinal disorders. We found that rectal swabs appeared to be a convenient means of sampling the human gut microbiota. Swabs can be performed on demand, whenever a patient presents; swab-derived microbiota profiles are reproducible, whether they are gathered at home by patients or by medical professionals in an outpatient setting and may be ideally suited for clinical diagnostics and large-scale studies.

## Introduction

Research into the composition of the gut microbiota in health and disease has bloomed since the advent of molecular approaches of its characterization. From the many investigations conducted in this field, evidence is accumulating that the composition of the gut microbiota may be related to disease states, most notably in inflammatory bowel diseases (IBD) [1], obesity [2] and malnutrition [3]. A prerequisite for using microbiota analysis as a clinical tool is efficient and consistent sampling and sample preservation [4]–[6]. An important factor in this regard is the influence of sample handling and the effect of intestinal preparation by bowel cleansing on composition of microbiota in stools and intestines [7], [8].

Currently, the most commonly used sample type for analysis of intestinal microbiota is feces. Classical microbial diagnostics on feces focuses on infectious gastroenteritis. For this purpose, current fecal sampling seems satisfactory. However, for a more comprehensive analysis of the intestinal microbiota, sampling, storage and processing of samples have a significant impact on the resulting composition analysis. The ideal sample should be easy to obtain in a standardized fashion with no preceding perturbation of the microbiota. Currently, fecal samples are usually collected by patients themselves at home. This introduces potential contamination during collection, time-lag before freezing, freezing above −20°C and thawing during transport to the laboratory. Evidence is compelling that these factors may introduce variation large enough to thwart microbial diagnostics [5]. An alternative is direct sampling of the intestinal mucosa by taking biopsies during endoscopy. While these samples can be gathered in a highly standardized fashion, the invasive nature of the sampling procedure precludes large-scale implementation as a screening or follow-up tool. In addition, prior to colonoscopy, patients are to be prepped with stringent laxative schemes, which have been shown to perturb the intestinal microbiota [8]. A third approach is to obtain samples by rectal swabbing. Rectal swabs can be easily obtained and can be stored immediately in a standardized fashion without previous perturbation of the microbiota. Rectal swabs are already regularly used for screening for resistant microbes and have shown to be very effective for that purpose [9].

In the present study we investigated the applicability of rectal swabs for gut microbiota analysis in a clinical routine setting. We analysed the effect of storage and processing conditions on reproducibility and compared microbial profiles by rectal swabbing to those from fecal and mucosal samples. Our results showed that rectal swabs are well suited for consistent and efficient routine sampling of the intestinal microbiota.

## Methods

### Design

**Figure 1:**
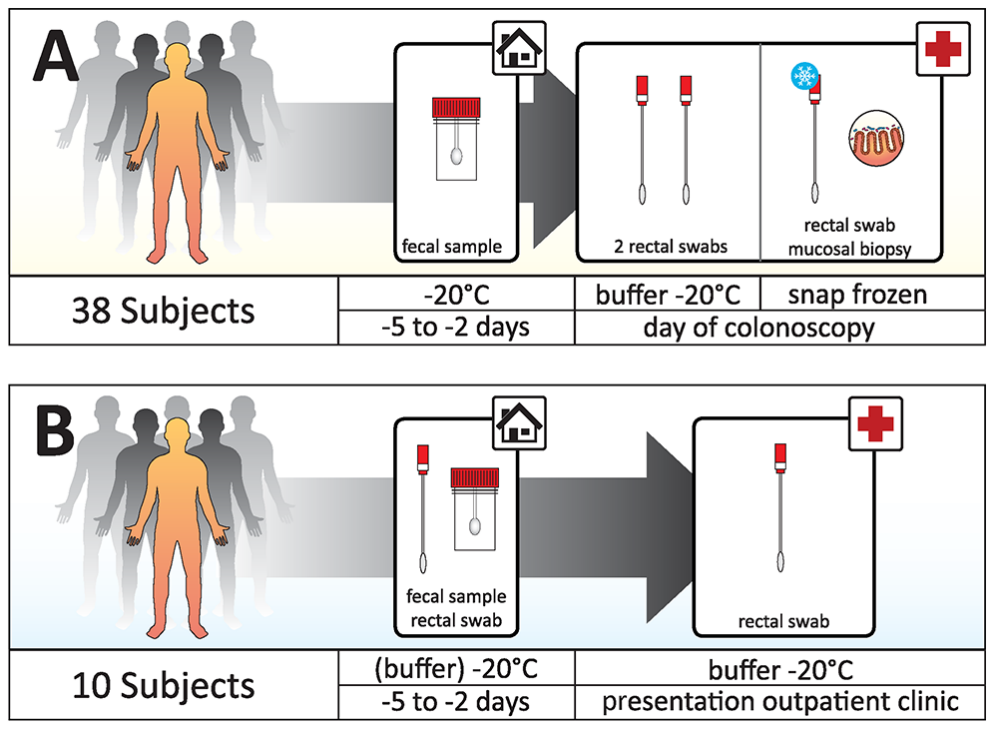
Two subject groups were sampled. Group A: 38 subjects who underwent an elective colonoscopy. Feces was collected at home two to five days before. Rectal swabs and biopsies were taken during the procedure. Two swabs were stored for two hours at room temperature and at −20°C afterwards, one swab was immediately snap frozen. The mucosal biopsy was washed in PBS and snap frozen. Group B: 10 patients with IBD. This group collected feces and one rectal swab at home. Feces was stored in a sterile container and the rectal swab in RTF buffer, both at at −20°C. A second rectal swab was obtained at the outpatient clinic and stored in the same fashion as the first swab.

This study was set up as a descriptive study to evaluate applicability and reproducibility of rectal swabs and to compare resulting bacterial profiles with those from feces specimens and mucosal biopsies. Two rounds of investigations were held. First, patients who underwent an elective colonoscopy were asked to bring in feces one week before the procedure and rectal swabs and biopsies were taken during the procedure. Because colonoscopy involves intestinal preparation, in a second round patients with inflammatory bowel disease who did not undergo intestinal preparation were asked to sample feces and to obtain one rectal swab at home; a second rectal swab was obtained on the day they brought in the feces (figure 1).

The study was approved by the institutional ethical review board of the VU University medical center in Amsterdam, NL, and all individuals provided written informed consent. Subjects were included who either underwent an elective colonoscopy between February and June 2011, or who presented for commonly scheduled control at the inflammatory bowel disease (IBD) outpatient clinic in October 2012. In the first group, the only exclusion criterion was a contraindication for taking mucosal biopsies.

### Samples

All patients who underwent colonoscopy were prepped according to a standardized protocol with a laxative preparation consisting of high-volume polyethylene glycol (PEG) solution (Moviprep, Norgine B.V., Amsterdam, The Netherlands). Mucosal biopsy specimens were collected with a flexible video endoscope (Olympus GmbH, Hamburg, Germany) and a flexible biopsy forceps (Wilson-Cook; European Endoscopy Group, Fujinon Medical Holland, Veenendaal, The Netherlands). Mucosal biopsy specimens were harvested from sigmoid colon at 20–30 cm from the anal verge. Per subject, one mucosal sample was washed twice in 500 µl PBS (pH 7) before snap-freezing in liquid nitrogen and a second sample was deposited in a container filled with 500 µl PBS and snap frozen in liquid nitrogen. All samples were stored at −20°C. In the colonoscopy group, rectal swabs were collected at the time of colonoscopy, just prior to the endoscopic procedure. Rectal swabs (FLOQSwabs 552C, Copan, CA, USA) were inserted into the anal canal, beyond the anal verge (±3 cm). For home swabbing, patients were instructed to do the same. Two rectal swabs were deposited in a container with 500 µl Reduced Transport Fluid (RTF) buffer [10] and kept at room temperature for 2 hours prior to storage at −20°C. One swab was immediately snap frozen in liquid nitrogen.

In the IBD outpatient group, two rectal swabs were gathered. One by the patients themselves at home, a day prior to presentation, the other was taken at the outpatient clinic. Both swabs were stored in a container with 500 µl RTF buffer at −20°C.

In both the colonoscopy as well as the IBD outpatient group, fecal samples were gathered within five days before presentation at the outpatient clinic. Samples were gathered in sterile containers and were stored at −20°C within 2 h after collection and kept frozen until further analysis. See Table S1 for a comprehensive listing of all samples.

### DNA isolation

DNA was isolated from feces or mucosal biopsies as described previously [11]. In short, for mucosal samples, the first step consisted of lysis of tissue and bacteria with the QIAamp DNA mini Kit (Qiagen, Hilden, Germany) followed by DNA extraction with the NucliSENS easyMag automated DNA isolation machine (Biomérieux, Marcy l'Etoile, France). For fecal samples, 100–400 mg of feces was used as input for the fecal DNA extraction protocol of the easyMag machine as described by the manufacturer. For DNA isolation from swabs, one ml of nucliSENS lysisbuffer, containing guanidine thiocyanate, was added to each vial containing a swab tip and the mixture was shaken at 1400 rpm (Thermomixer comfort, Eppendorf, Hamburg, Germany) for five minutes. For a subset of 14 snap frozen swabs, an additional bead-beating step was evaluated. For these swabs, after five minutes of shaking at 1400 rpm, the mixture was divided into two parts. To one part, approximately 100 µg of Zirconia 0.1 silica beads were added and bead-beating was performed for 60 seconds. Afterwards, all samples were centrifuged for four minutes at 12.000 g and added to the easyMag container. DNA extraction was performed on the easyMag machine with the Specific A protocol as described by the manufacturer.

### IS-profiling of the intestinal microbiota

The intestinal microbiota analysis was performed by IS-pro as described previously [11]. IS-pro involves bacterial species differentiation by the length of the 16S–23S rDNA interspace region with taxonomic classification by phylum-specific fluorescent labelling of PCR primers.

#### Amplification of IS regions

Five primers were used for amplification of IS regions. Two fluorescently labelled forward primers were phylum-specific, for the 16S rDNA region: one FAM-labelled primer, specific for *Firmicutes*, *Actinobacteria*, *Fusobacteria and Verrucomicrobia* (for efficiency this group will further be referred to as *AFFV*) and one HEX-labelled primer specific for *Bacteroidetes*. Three unlabelled reverse primers were specific for the 23S rDNA region. The combination of these primers provided very broad coverage for *Firmicutes*, *Actinobacteria, Fusobacteria, Verrucomicrobia*, and *Bacteroidetes*. The primers were used in a multiplex PCR, which amplified the 16S–23S IS region. The length of this IS region and its PCR product is species-specific. The fluorescent label provides identification of all fragments at the phylum level.

Amplifications were carried out on a GeneAmp PCR system9700 (Applied Biosystems, Foster City, CA). Cycling conditions for PCR were 72°C for 2 min; 35 cycles of 94°C for 30 s, 56°C for 45 s, and 72°C for 1 min; and a final extension at72°C for 5 min. Each PCR mixture, with a final volume of 25 µl, contained 10 µl of buffered DNA, 1x superTaq buffer (SphaeroQ, Gorinchem, the Netherlands), 200 µM deoxynucleoside triphosphate, 0.04% BSA, 1 U of superTaq, and 0.13 µM of each of the 5 primers.

#### IS-Fragment analysis

**Figure 2:**
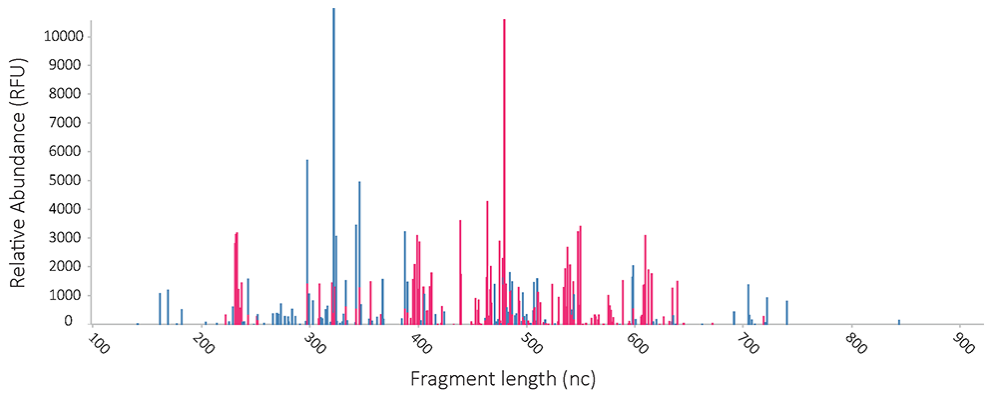
Sample IS-profile. The x-axis represents IS fragment length, the y-axis represents relative abundance of fragments. Colours of fragments correspond to bacterial phyla. Blue peaks represent *AFFV group*, red peaks represent *Bacteroidetes*. Each peak may be regarded as an operational taxonomic unit (OTU).

After PCR, 5 µl of PCR product was mixed with 19.8 µl formamide and 0.2 µl Mapmaker 1000 ROX-labeled size marker (BioVentures, Murfreesboro, TN, USA). DNA fragment analysis was performed on an ABI Prism 3130XL Genetic Analyzer (Applied Biosystems). Results are presented as color-labeled peak profiles (figure 2). These peaks can be regarded as operational taxonomical units (OTU's). All data were further analyzed with the Spotfire software package (TIBCO, Palo Alto, CA, USA). All raw data is available in Table S2.

### Statistical analysis

#### Repeatability and reproducibility

Variations in peak heights by repeated testing, repeated sampling or different storage methods were quantified by means of a variance components analysis. For twelve rectal swabs, the lysis buffer that was added to the swab tip was split into two aliquots and processed separately. From the resulting profiles, repeatability ε was estimated according to the model:
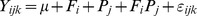



The variation introduced by repeated sampling was estimated from 29×2 rectal swab profiles from the two rectal swabs that were taken just prior to the endoscopic procedure and stored in RTF at room temperature for two hours. Sampling variation σ was estimated according to the model:
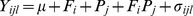



Variation introduced by storage at room temperature was estimated from 29×2 rectal swabs of which one was stored in RTF at room temperature for two hours and the other was directly snap frozen in liquid nitrogen. Storage variation τ was estimated according to the model:
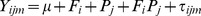



In all cases, the response variable *Y* is the log2 transformed peak value within a one nucleotide interval for a particular fragment *i* as measured in a rectal swab profile taken from patient *j*. Furthermore, *μ+F_i_* denotes the average overall response in the study population for a particular fragment *i, P_j_* is the average overall deviation for the *j*th patient and *F_i_P_j_* is the additional deviation on the *i*th fragment for the *j*th patient. Finally, the factors *ε_ijk_*, *σ_ijl_* and *τ_ijm_* are the deviation from the *j*th patient mean for the *i*th fragment on the *k*th repeated sample, the *l*th duplicate sample and the *m*th storage method, respectively.

We only incorporated those fragments that yielded a meaningful response (defined as a peak value within a one nucleotide interval above or equal to 128 relative fluorescence units (RFU)) in both duplicates [11]. Because the number of fragments is limited by the nature of the assay, *F_i_* was modelled as a fixed effect. All other terms were modelled as random effects. Computations were performed with the ML method using the SAS procedure VARCOMP.

### Correlation of profiles

Correlation analyses were performed as described previously [11]. Comparisons between all samples were made by calculating squared correlation coefficients for all possible pairs of samples. When duplicate swabs stored in RTF from the colonoscopy group were compared to other samples, averaged profiles of the duplicate swabs were used.

Comparisons were grouped in intra and inter individual comparisons, the former group comprising all comparisons between samples from the same individual, the latter group comprising all other comparisons. Median and inter quartile range (IQR) were calculated for each group.

### Diversity analysis

Diversity was calculated per phylum and for the overall microbial composition by pooling all phyla together. Within-sample (alpha) diversity was calculated as the Shannon index, which was recently shown to be a robust estimate of microbial diversity [12]. Diversity indices were calculated for all sample types from both the colonoscopy and the IBD outpatient group. Dissimilarities between samples, or between-sample diversity, were represented in a dissimilarity matrix that was built using the cosine distance measure. Given two vectors of attributes (two profiles in our case), A and B, the cosine dissimilarity is represented using a dot product and magnitude as:
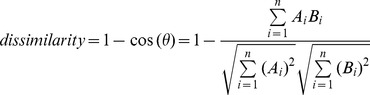



The resulting dissimilarity matrix was summarized and visualized in a low-dimensional space using principal coordinate analysis (PCoA). Diversity analysis was performed using the vegan software package in R.

## Results

### Study population

A total of 38 subjects was included in the colonoscopy group, 23 male and fifteen female. The indications for colonoscopy were suspected neoplasm (8), screening for familial tumours (5), IBD (5), and general gastrointestinal complaints (13), of which diverticulosis was the most common diagnosis (6 out of 13). Not all sample types could be harvested from all subjects and in a few samples the PCR reaction was inhibited. The total number of samples obtained was: 35 snap frozen rectal swabs, 37 sets of rectal swabs in RTF, 33 dry mucosal biopsies, 35 mucosal biopsies in sodium chloride 0.9% and 19 fecal samples.

Ten subjects were included in the IBD outpatient group, two male (both with ulcerative colitis) and eight female (four with ulcerative colitis, four with Crohn's disease). Two subjects presented with active disease, eight with disease in remission. The total number of samples was: ten swabs and ten fecal samples taken at home by the patients themselves and ten swabs taken at the outpatient clinic by their physician.

### Effect of bead-beating for DNA isolation

**Figure 3:**
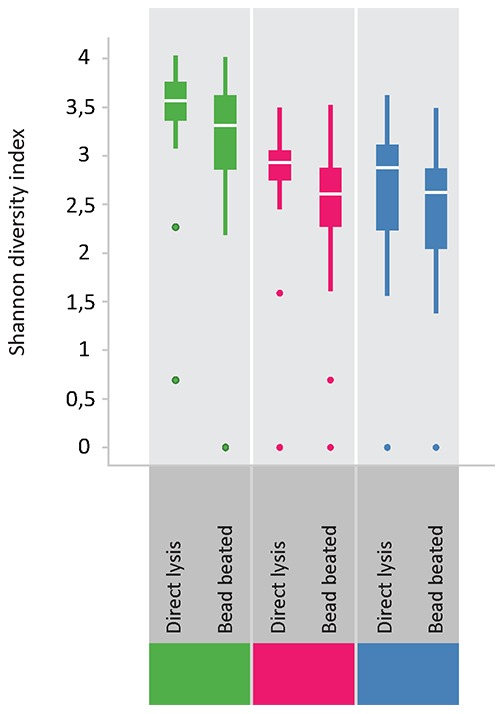
Diversity analysis of samples that were either directly lysed or underwent bead-beating prior to DNA isolation. Shannon diversity indices are generally lower for bead-beated samples for the phylum Bacteroidetes (pink), the AFFV group (blue) and consequently for all phyla combined (green).

As it has been described that DNA isolation protocols for fecal samples that include bead-beating give higher DNA yields of certain groups of bacteria [13], we compared this procedure to automated DNA isolation for swab samples. With a mixed effects model, accounting for both fixed and random effects, we found bead-beating to have a significant negative impact on DNA recovery from *Bacteroidetes*. *Bacteroidetes* peaks in DNA isolated without a bead beating step were on average 1.85 times higher than the equivalent peaks in DNA isolated with a bead beating step (p = 0.015). For the *AFFV* group there was also a trend towards a negative impact of bead beating. Peaks from DNA without bead beating were on average 1.45 times higher than equivalent peaks generated from DNA with a bead-beating step (p = 0.051). Furthermore, bead-beating also showed a negative impact on the estimated diversity both for *Bacteroidetes* and the AFFV group (figure 3).

### Effect of storage and processing of rectal swabs

**Figure 4:**
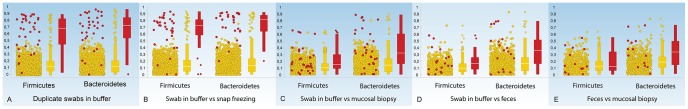
Comparisons of microbiota profiles of the colonoscopy group expressed as R squared. All comparisons have been done separately for *AFFV* group (left) and *Bacteroidetes* (right). Figures show comparisons of all profiles. Red dots represent comparisons of samples of the same subject (intra-subject correlation). Yellow box plots are based on all correlations, red box plot on intra-subject correlations only. A: Duplicate swab profiles stored in RTF buffer. B: Swab stored in RTF buffer versus snap frozen swabs. C: Swabs stored in buffer versus mucosal biopsies. D: Swabs stored in buffer versus fecal samples. E: fecal samples versus mucosal biopsies.

To evaluate the effect of a non-stringent sample storage protocol, we compared microbiota profiles of snap-frozen rectal swabs to profiles of rectal swabs that had been stored at room temperature in RTF for two hours before freezing at −20°C. As storage conditions may affect Gram-positive and Gram-negative bacteria differently, we analyzed data separately for *AFFV* and *Bacteroidetes*. Correlations were calculated for profiles derived from snap frozen swab samples versus the averaged profiles of duplicate swab samples stored in RTF at room temperature before freezing. For both *AFFV* and *Bacteroidetes*, intra-individual comparisons showed high similarities as measured by a high median R^2^. Inter individual comparisons were low, as was expected (figure 4B). For all measured values in the above and subsequent sections, we refer to Table 1.

**Table 1:** Median R squared and Inter Quartile Range (IQR) values for all comparisons.

Group	Comparator 1	Comparator 2		AFFV	Bacteroidetes
**A (colonoscopy)**	Swab	Snap frozen swab	A	0,73 (0,23)	0,81 (0,24)
	Swab	Snap frozen swab	B	0,12 (0,16)	0,13 (0,20)
	Swab	Duplicate swab	A	0,70 (0,38)	0,72 (0,31)
	Swab	Duplicate swab	B	0,13 (0,15)	0,12 (0,17)
	Swab	Faeces	A	0,17 (0,18)	0,36 (0,35)
	Swab	Faeces	B	0,10 (0,12)	0,16 (0,20)
	Swab	Mucosal biopsy	A	0,15 (0,21)	0,32 (0,44)
	Swab	Mucosal biopsy	B	0,10 (0,11)	0,13 (0,19)
	Faeces	Mucosal biopsy	A	0,12 (0,28)	0,33 (0,35)
	Faeces	Mucosal biopsy	B	0,12 (0,14)	0,18 (0,16)
**B (IBD)**	Swab at home	Swab (hospital)	A	0,55 (0,26)	0,82 (0,23)
	Swab at home	Swab (hospital)	B	0,14 (0,03)	0,03 (0,14)
	Swab at home	Faeces	A	0,23 (0,52)	0,75 (0,39)
	Swab at home	Faeces	B	0,10 (0,12)	0,05 (0,20)

IQR values are indicated in brackets. Rows A and B indicate either intra-individual comparisons (A) or inter-individual comparisons (B).

### Repeatability and reproducibility

The estimated variance for repeated testing of same samples was 0.25 log2RFU for *Bacteroidetes* and 0.37 log2RFU for *AFFV*. Total sampling variance, which includes variance introduced by repeated sampling as well as repeated testing, was estimated to be 0.78 log2RFU for *Bacteroidetes* and 1.16log2RFU for *AFFV*. Storage variance, introduced by storing swabs at room temperature in RTF buffer instead of direct snap freezing, was estimated to be 0.79 for *Bacteroidetes* and 1.14 for *AFFV*. Thus, additional variance was introduced by taking multiple samples as compared to repeated testing of the same sample. However, no additional variance was introduced by storing swabs at room temperature in RTF for two hours as compared to directly snap freezing them in liquid nitrogen.

### Correlation of swab derived profiles

To further evaluate the reproducibility of the swabbing procedure, we performed a correlation analysis on swabs taken from the same patient (n = 37). We compared the duplicate swabs that were stored in RTF buffer at room temperature for 2 hours with each other and with the swabs that were directly snap frozen. Log 2 transformed profiles were compared pairwise intra- and inter individually with Pearson correlation and results were analyzed separately for *Bacteroidetes* and *AFFV*. For the duplicate swabs stored in RTF, intra-subject correlations were high for both *AFFV* and *Bacteroidetes*, whereas inter individual correlations were low. Correlations between RTF-stored swabs and snap frozen swabs were also very high for both *Bacteroidetes* and *AFFV* (Figure 4A and 4B). These data underline the reproducibility of rectal swabs within the same patient, regardless whether samples are stored in RTF buffer at room temperature for two hours or are directly snap frozen in liquid nitrogen.

**Figure 5:**
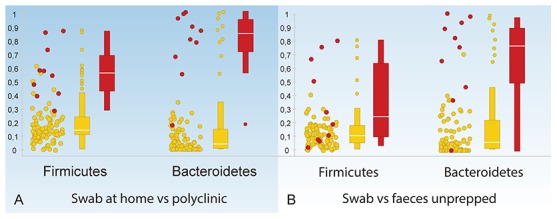
Comparisons of microbiota profiles in the IBD outpatient group expressed as R squared. All comparisons have been done separately for *AFFV* group (left) and *Bacteroidetes* (right). Figures show comparisons of all profiles. Red dots represent comparisons of samples of the same subject (intra-subject correlation). Yellow box plots are based on all correlations, red box plots on intra-subject correlations only. A: Swabs taken by patients at home versus swabs taken by the physician at the polyclinic. B: Swabs versus fecal samples.

To test whether self-sampling by patients at home would yield comparable results to swabs taken at the outpatient clinic, we compared these in ten individuals. Again, profiles were compared pair wise and results were analyzed separately for *AFFV* and *Bacteroidetes*. This analysis too showed high intra-subject correlations and low inter-subject correlations (Figure 5A). This showed that self-collected swabs were highly comparable to clinically collected swabs when stored in the same fashion.

### Correlation of different sample types

Finally, rectal swabs, fecal samples and mucosal biopsies were compared by correlation of profiles. First, we compared swab samples to mucosal biopsy samples taken by colonoscopy in 32 subjects. These correlations were generally low, especially for the *AFFV* group. *Bacteroidetes* profiles showed a somewhat higher similarity with a large distribution of values for R^2^ (Figure 4C). Next, rectal swab profiles were compared to fecal profiles in nineteen subjects of the colonoscopy group and in all ten subjects of the IBD outpatient group. In the colonoscopy group, correlations between swab and fecal profiles were generally low, similar to the correlation of swab samples to mucosal biopsy samples in this prepped patient group. *Bacteroidetes* generally had a slightly higher correlation than *AFFV* (Figure 4D). In the IBD outpatient group, correlations were markedly higher, especially for the *Bacteroidetes*, which had a median R^2^ similar to that found for duplicate swab profiles (Figure 5B). As expected, inter subject correlations were low in both groups. These data showed that fecal profiles resembled swab profiles, in particular for the phylum *Bacteroidetes*, but not in people who underwent bowel prepping.

Finally, as we found rectal swab microbiota profiles to be distinct from mucosal and fecal microbiota profiles in the colonoscopy group, we were interested in the similarity between fecal microbiota and mucosal microbiota. To compare these, we used the same analysis as above. We found that correlations between fecal samples and mucosal biopsies were as low as the correlations of swab samples to both these sample types. Again, a higher correlation was found for *Bacteroidetes* than for *AFFV* (figure 4E).

### Diversity analysis

**Figure 6:**
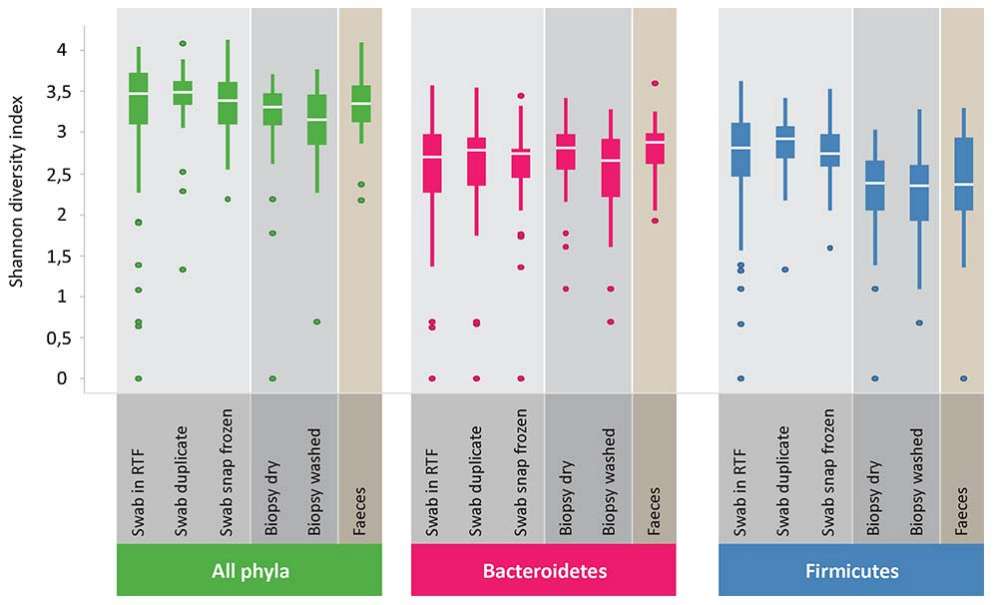
Diversity analysis of the different sample types. Shannon diversity indices are highly similar between duplicate swabs and snap frozen swabs for all phyla. Diversity is lower for the *AFFV* group in mucosal biopsies and fecal samples compared to rectal swabs.

**Figure 7:**
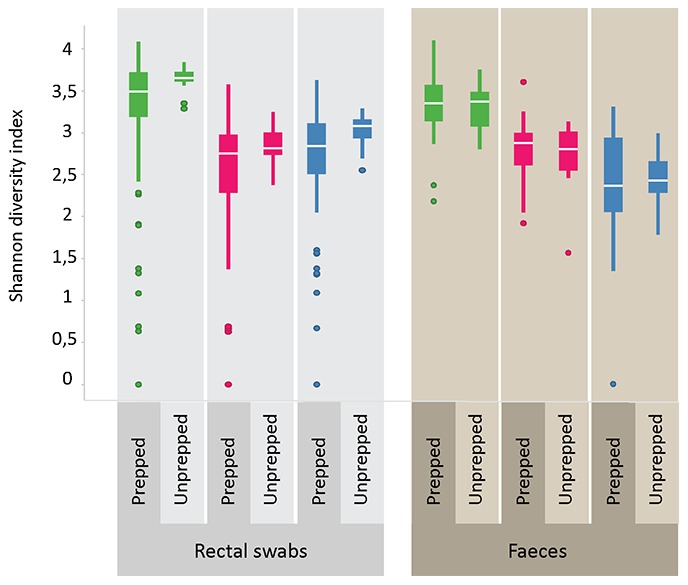
Comparisons of microbial diversity in prepped versus unprepped subjects. Diversity in fecal samples is similar in both groups. For rectal swab samples, diversity seems to be somewhat higher in the unprepped group, and distribution of diversity indices are somewhat smaller than in the prepped group. *AFFV* diversity can be seen to be higher in rectal swabs than in fecal samples.

**Figure 8:**
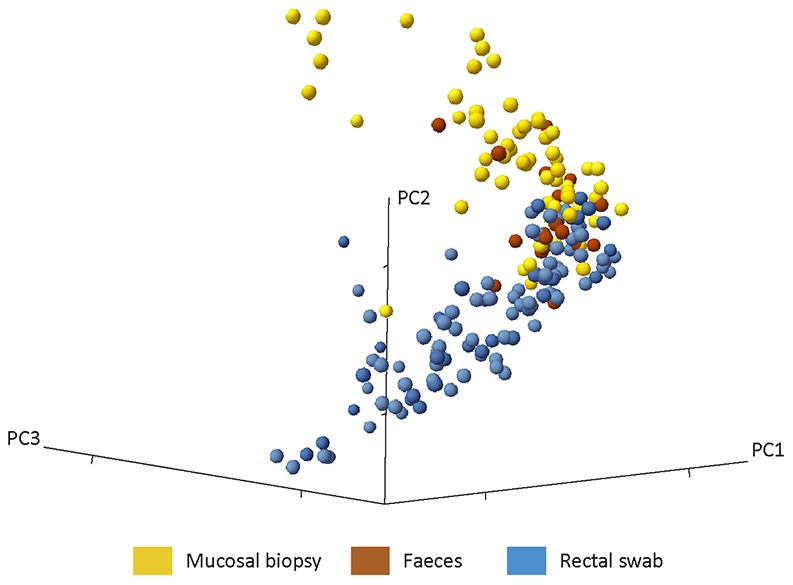
Principal coordinate analysis depicting the different sample types. Rectal swabs, fecal samples and mucosal biopsies may sometimes be very similar, but on a whole, these three sample types seem to harbor more or less distinct microbiota.

Shannon diversity indices were highly similar between duplicate swabs and snap frozen swabs for all phyla. The most pronounced difference in diversity indices for the various sample types was in the *AFFV* group. Here, diversity was markedly lower in mucosal biopsies and fecal samples than in rectal swabs. For the phylum *Bacteroidetes*, diversity was similar for the different sample types (figure 6). Furthermore, we compared microbial diversity in prepped versus unprepped individuals for rectal swabs and fecal samples. As fecal samples were taken before bowel prepping, we did not expect to find differences in diversity in this sample type between the two groups. Indeed, diversity in fecal samples was very similar in both groups. For the rectal swab samples, diversity seemed to be somewhat higher in the unprepped group, and distribution of diversity indices appeared smaller than in the prepped group. Moreover, the higher diversity in rectal swabs as compared to fecal samples held true for both the prepped as well as the unprepped group (figure 7). A comparison between all sample types was made with a cosine dissimilarity matrix and visualized by principal coordinate analysis. From this analysis it becomes apparent that rectal swabs, fecal samples and mucosal biopsies may sometimes be very similar, but that on a whole, these three sample types seem to harbor more or less distinct microbiota (figure 8).

## Discussion

In this study we showed that rectal swabs provide a good method to produce highly reproducible microbiota profiles. Profiles are similar to those of fecal samples in unprepped patients, especially for the phylum *Bacteroidetes*. We suggest that rectal swabs may be ideally suited for large-scale studies and for routine clinical applications of microbiota profiling. Whereas correlation of swab profiles to fecal profiles was quite high in patients who did not undergo extensive intestinal preparation for colonoscopy, the similarity between different sample types was markedly lower in the colonoscopy group. Most likely, this was due to the preparatory bowel lavage, which obviously affected the intestinal content. Therefore, we suggest that sampling of the intestinal microbiota with feces or by rectal swabbing, without previous bowel preparation, is probably the preferred method if analyzing genuine, undisturbed microbiota. Rectal swabs are an attractive means of sampling the intestinal microbiota in a clinical setting without the drawbacks of feces collection or mucosal sampling. Subjects do not need to collect fecal samples at home and do not need to be prepped or undergo invasive procedures. The applicability of rectal swabs is highlighted by the fact that they are already commonly used in clinical routine and can be taken at every visit.

We further showed that short-term storage of rectal swabs in RTF buffer at room temperature had no impact on the composition of the microbiota, thus relaxing requirements for sample collection and storage and making the method applicable in almost any (clinical) setting. The effects of various storage protocols on microbiota composition have been investigated previously. Prolonged storage of feces at room temperature as well as thawing of feces >1 hour before DNA isolation has been shown to impact the microbiota composition [5]. It has also been found that storage at room temperature impacts microbiota analysis on feces [4], [6]. In contrast, it has also been reported that storage of feces has no significant effect on microbiota composition [14]. This lack of effect seems improbable, since analysis by classic culture has amply shown that storage of feces at room temperature leads to marked changes in microbial composition. Regardless of these discrepancies, we here demonstrated that storage of rectal swabs at room temperature for 2 hours in a stabilizing buffer (RTF buffer), did not impact microbiota composition.

As bead-beating has been described to be of added value in DNA extraction especially for bacteria in the phylum *Firmicutes* [7], [15], we evaluated this for swab samples. In these samples it did not contribute to the DNA yield. In contrast, bead-beating diminished the yield of *Bacteroidetes* DNA. This was an unexpected, yet reproducible outcome in the context of what has been described for isolation of bacterial DNA from feces. As composition of microbiota in rectal swabs does not differ markedly from that in fecal samples –especially in *Bacteroidetes*-, composition cannot have been a factor. We hypothesize that the effect may be due to the large differences in bacterial loads in fecal and swab samples. With lower loads, bead beating may damage DNA, instead of contributing to DNA yield. For clinical routine, in which speed and ease is essential, omission of bead beating is favorable.

We have performed repeatability and reproducibility analysis previously on mucosal biopsies and fecal samples [11]. The values as measured here for rectal swabs are highly similar to those previously reported findings. The practical implication of this is that sampling variation in microbiota profiles introduced by the swabbing procedure itself is no larger than that introduced by taking multiple mucosal biopsies or by analyzing different sub samples of the same fecal sample. Reproducibility of rectal swab samples was further underlined by high total profile correlations and similarity of diversity indices.

It has been shown that the microbiota composition of rectal swab samples is similar to that of fecal samples and less similar to the microbiota composition of mucosal biopsies in a group of patients with colorectal carcinoma [16]. This similarity was believed to be due to adherence of feces to swab samples, since they harvested the swabs from patients that were not prepped for colonoscopy. In these series, we showed that swab profiles are indeed similar to feces profiles obtained from unprepped subjects -in particular for the phylum *Bacteroidetes*-, but decidedly distinct in prepped patients. In these prepped subjects, microbiota profiles in swab samples were also distinct from profiles in mucosal biopsies. Rectal swabs were taken just prior to colonic mucosal biopsies, both after prepping of the subject. The difference in composition between these samples thus seemed to represent a true difference in composition between the proctum, which was sampled by rectal swabs, and more proximal in the (distal) sigmoid colon, as sampled by sigmoidal mucosal biopsies. The higher diversity of the *AFFV* group in rectal swab profiles may be caused by the presence of a different array of species characteristic for the transitional zone of a strict anaerobic to a more aerobic environment. Also, the stratified squamous epithelium characteristic for the lower part of the anal canal may support different microbial species than the columnar epithelium of the more proximal parts of the colon. In this context it is interesting to note that ulcerative colitis always commences at exactly this transitional zone from whereon the disease proceeds inward.

The study presented here has been specifically set up to evaluate feasibility and reproducibility of a convenient sampling method for intestinal microbiota profiling that can easily be used in a routine clinical setting. Rectal swabs indeed proved very convenient. However, there are some potential drawbacks of rectal swabs, such as unwillingness of patients to undergo the swabbing procedure because of discomfort, a potential lack of biomass captured by the swab and the potential of contamination of the sample with skin bacteria. Concerning patient compliance, we found that patients were generally willing to undergo the procedure after a brief explanation. As of the lack of biomass, we did not find evidence of this theoretical problem in our study. Also, we did not find evidence of contamination by skin bacteria. We hypothesize that extent of this potential problem is limited, as bacterial load in the rectum is orders of magnitude higher than on the skin.

We applied strict *per protocol* execution for all procedures in this study to be able to evaluate the impact of isolated effects on microbiota analysis. There are yet some additional aspects that would have been interesting to evaluate, like evaluating more storage regimens or different DNA isolation methods. However, this would have required taking more swabs than the three that were harvested for this study. Moreover, the EasyMag system for DNA isolation as was employed here, has been described to be very suitable for extraction of DNA from fecal samples. Therefore only preprocessing with bead-beating was tested as a variable, as this has been described to be of added value in fecal samples.

In conclusion, it is important to define standards for reproducible and accurate sampling of gut microbiota that can be implemented in clinical routine.We found that rectal swabs were a convenient means of sampling the human gut microbiota. Swabs can be taken on demand, whenever a subject presents. The acquired samples resembled fecal microbiota and showed a highly reproducible profile, whether they were gathered at home by patients or by medical professionals in an outpatient setting.

## Supporting Information

Table S1 **Listing of all samples per patient.** All samples are listed here per patient, with information on sample type and DNA isolation method. The last column (Sample_ID) may be used to link this information to the information in Table S2.
(TXT)

Table S2 **Raw data of all samples.** Here raw data for all analyzed samples is provided. Each line represents a specific fragment length for a specific sample for a particular primer set with the corresponding intensity of that fragment.
(TXT)
